# Track Structure Study for Energy Dependency of Electrons and X-rays on DNA Double-Strand Break Induction

**DOI:** 10.1038/s41598-019-54081-6

**Published:** 2019-11-27

**Authors:** Yoshie Yachi, Yuji Yoshii, Yusuke Matsuya, Ryosuke Mori, Joma Oikawa, Hiroyuki Date

**Affiliations:** 1 Graduate School of Health Sciences, Hokkaido University, Kita-12 Nishi-8, Kita-ku, Sapporo, Hokkaido 060-0812 Japan; 2 Biological Research, Education and Instrumentation Centre, Sapporo Medical University, Minami-1 Nishi-17, Chuo-ku, Sapporo, 060-8556 Japan; 3 Japan Atomic Energy Agency (JAEA), Nuclear Science and Engineering Centre, Research Group for Radiation Transport Analysis, 2-4 Shirakata, Tokai, Ibaraki 319-1195 Japan; 4 Department of Radiology, Tokyo University Hospital, Tokyo, 113-8655 Japan; 5 Faculty of Health Sciences, Hokkaido University, Sapporo, 060-0812 Japan

**Keywords:** Cell death, Atomic and molecular physics

## Abstract

Radiation weighting factor *w*_*R*_ for photons and electrons has been defined as unity independently of the energy of the particles. However, the biological effects depend on the incident energies according to *in vitro* experimental data. In this study, we have quantified the energy concentration along electron tracks in terms of dose-mean lineal energy (*y*_*D*_) on chromosome (micro-meter) and DNA (nano-meter) order scales by Monte Carlo simulations, and evaluated the impact of photon energies on DNA double-strand break (DNA-DSB) induction from an experimental study of irradiated cells. Our simulation result shows that the *y*_*D*_ values for diagnostic X-rays (60–250 kVp) are higher than that for therapeutic X-rays (linac 6 MV), which agrees well with the tissue equivalent proportional counter (TEPC) measurements. The relation between the *y*_*D*_ values and the numbers of γ-H2AX foci for various photon energy spectra suggests that low energy X-rays induce DNA-DSB more efficiently than higher energy X-rays even at the same absorbed dose (e.g., 1.0 Gy). The relative biological effectiveness based on DNA-DSBs number (RBE_DSB_) is proportionally enhanced as the *y*_*D*_ value increases, demonstrating that the biological impact of the photon irradiation depends on energy concentration along radiation tracks of electrons produced in the bio-tissues. Ultimately, our study implies that the value of *w*_*R*_ for photons varies depending on their energies.

## Introduction

Incidents of photon beams (i.e., X-rays and γ-rays) on biological tissue give rise to interactions with materials, especially generations of secondary electrons via photoelectric absorption and Compton scattering, etc^1^. Secondary electrons deposit their energies into cells in the tissue through collision processes such as ionization, which may induce DNA damage leading to chromosomal aberrations and cell death^2^. For making clear the biological effectiveness of photons on intercellular materials, it is crucial to analyse the local energy deposition of secondary electrons on micro- or nano-meter scale^3^.

As an index of the biological effectiveness of ionizing radiation, the radiation weighting factor (*w*_*R*_) has been used extensively^4^. In the International Commission on Radiological Protection (ICRP) 60 publication, the *w*_*R*_ value for low linear energy transfer (LET) radiation represented by photons and electrons is defined as 1.0, independent of radiation energy^4^. However, at the endpoint of cell damage, caused by DNA double-strand breaks (DNA-DSBs), lower energy photon beams induce γ-H2AX foci (indicating more DNA-DSBs) than higher energy beams^5^. In addition, it has been well established that δ-rays (secondary electrons created by primary ionizing radiation) have a large impact on bio-tissues^6^. The relative biological effectiveness (RBE), specifically RBE based on DNA-DSBs (RBE_DSB_)^6,7^ and RBE on 10% cell survival (RBE_10_)^7^, has so far been noted to depend on photon energy by *in vitro* biological experiments. Further investigation for biological impact is necessary for determining RBE and *w*_*R*_ for various photon energies ranging from diagnostic to therapeutic.

LET has been used as an indicator to evaluate the quality of ionizing radiation, which represents the energy transferred per unit length of track^8,9^. Whilst LET is given by the average dose over the track length, microdosimetry^10^ tries to obtain stochastic quantities related to chromosome and DNA within the micro-size volume of affected structures^10^. The induction yield of DNA-DSBs after exposure to electrons and photons has been discussed in previous studies^11,12^. For confirming the dependency of RBE on the photon energy as noted in biological observations, further investigations of the cells exposed to various energy spectra of electrons and photons, particularly on the characteristics of local energy deposition along the track, are necessary.

In this study, we focused on the DNA damage induction and the energy depositions of electrons (created by X-rays) at submicron scales by the use of Monte Carlo simulation techniques and by conducting immunofluorescent staining experiments with cultured cells. From the relation between the γ-H2AX foci induction and the dose-mean lineal energy (*y*_*D*_) value, we can see the impact of electron and photon energies on RBE in more detail. The results show a well-marked tendency that lower energy (diagnostic) X-rays are more effective for DNA damage than higher energy (therapeutic) X-rays.

## Materials and Methods

### Sampling technique of lineal energy along the electron track

**Figure 1:**
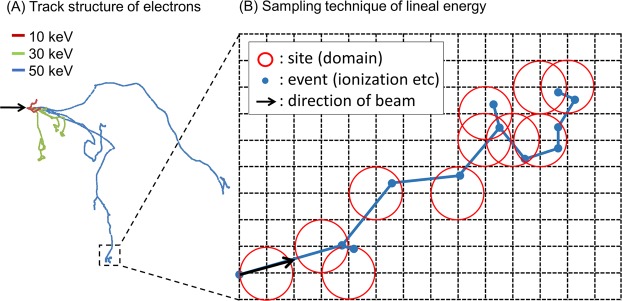
Schematic illustration of the technique for sampling the energy deposition by electrons: (**A**) is for the track structure of electrons with 10, 30 and 50 keV simulated by the WLTrack code^13^, and (**B**) is for the sampling technique to calculate the lineal energy, *y*_*D*_ (keV/μm). To calculate the *y*_*D*_ value, we took two steps: (1) select scoring positions (indicated by blue dots) on a grid pattern considered for electron track structures (indicated by a dotted black line), (2) set sampling sphere site (indicated by red circles) centering on sampling positions and score the energy deposition in each site. The radius of the sphere is the same as the grid size.

We used an in-house Monte Carlo code “WLTrack”^13^ for calculating the track structure of electrons (Fig. 1A). We developed a random sampling technique for energy deposition on the micrometer scale along the electron track. The sampling sites are placed uniformly along an electron track using a similar technique as the sampling method for lineal energy reported by Famulari, *et al*.^3^. A schematic representation of this technique is illustrated in Fig. 1B. After the sampling of energy deposition in the site volume, we calculated the lineal energy *y* in keV/µm according to ICRU report 36^10^ by

1 $$y=\frac{\varepsilon }{\mathop{l}\limits^{-}}.$$

Here, *ε* is the energy deposited in the volume of the sampling site and $\bar{l}$ is the mean chord length of the site volume defined by $\bar{l}$ = 4*r*_d_/3 (*r*_d_ is the radius of the site sphere). In consideration of the probability density of dose as a function of lineal energy, *d*(*y*), the dose-mean lineal energy *y*_*D*_ in keV/µm is given by

2 $${y}_{D}=\int yd(y){\rm{d}}y\,=\,\frac{\int {y}^{2}f(y){\rm{d}}y}{\int yf(y){\rm{d}}y},$$

where *f*(*y*) is the probability density of lineal energy *y*. We calculated the *y*_*D*_ value for mono-energetic electron and photon beams with various energy spectra to evaluate the radiation quality.

### Calculation of dose-mean lineal energy for electrons and photons

To validate the sampling technique used in this study, the *y*_*D*_ values of mono-energetic electrons in the kinetic energy ranging from 0.1 to 1000 keV were calculated and compared with a previous report by Famulari, *et al*.^3^. In our simulation for calculating *y*_*D*_, we considered five interaction processes: ionization, electronic excitation, elastic scattering, attachment and vibrational excitation. The number of electrons traced for every initial mono-energy was 10^2^. The cut off energy for the simulation was set 1.0 eV.

**Figure 2:**
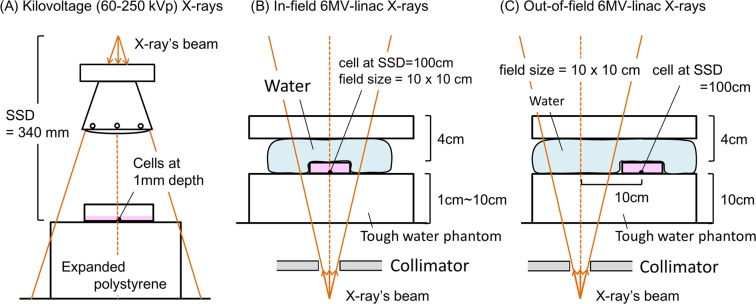
The geometries of various X-ray experiments: (**A**) for kilo-voltage X-rays such as 60 kVp, 100 kVp, 200 kVp, 250 kVp, (**B**) for in-field irradiation with 6 MV linac X-rays at various depths from the surface (1 cm-10 cm), and (**C**) for out-of-field irradiation with 6 MV linac X-rays at 10 cm depth from the surface. Cultured CHO-K1 cells in plateau phase were located at the isocenter and exposed to 1 Gy of X-rays.

The *y*_*D*_ value for photon irradiation was calculated by using two Monte Carlo codes, Particle and Heavy Ion Transport code System (PHITS) ver. 3.02^14^ for photons (cutoff = 1.0 keV) and WLTrack for electrons (cutoff = 1.0 eV). The geometries for various irradiations of 60 kVp, 100 kVp, 200 kVp, 250 kVp and 6 MV-linac X-rays are described in Fig. 2. In the irradiation of 6 MV linac X-rays, the percentage dose depth (PDD) was set to be 1, 3, 5, 10 cm for “in-field” irradiation, while PDD was fixed at 10 cm for “out-of-field” irradiation (10 cm away from isocenter). The procedure for calculating the *y*_*D*_ value is summarized as follows:

(i) Calculate the electron energy spectra for photon irradiation using PHITS^14^,
(ii) Sample the energy deposition along the electron tracks using WLTrack^13^,
(iii) Calculate the *y* and *y*_*D*_ values based on Eqs. (1) and (2).

To validate the sampling algorithm, we calculated *y*_*D*_ values for 200 kVp X-rays and 6 MV linac X-rays at 10 cm depth from the surface. The results were compared with the measurement values from Tissue Equivalent Proportional Counter (TEPC) reported by Okamoto, *et al*.^15^. After the confirmation of agreement between our results and the measured data^15^, we proceeded to the *y*_*D*_ value calculations for various X-rays energy spectra at the energies of diagnostic and therapeutic X-rays for evaluating the dependency of X-ray energy on DNA damage induction (DNA-DSBs). All calculations were conducted three times and the mean value with its uncertainty was estimated.

### Cell line and cell culture

To evaluate the biological effects by various photon irradiations, we adopted the Chinese Hamster Ovary (CHO)-K1 cell line, obtained from RIKEN Bio Resource Center, Japan (RCB0285). The CHO-K1 cells were maintained in Dulbecco’s Modified Eagle’s Medium-high glucose (D5671 Sigma) supplemented with 10% fetal bovine serum (Equitech-Bio Inc, Kerrville, TX) at 37 °C in a humidified 95% air and 5% CO_2_ incubator. The ϕ12-mm glass-based dishes (3911–035, IWAKI) were used for all irradiations. The cells were seeded onto the glass (0.08–0.12 mm in thickness) of the dish. The cells in plateau phase (mainly in G_1_ phase) were prepared for all *in vitro* experiments of DNA-DSBs’ detection as reported previously^16,17^.

### Irradiation setup

We employed five types of X-rays: 60 kVp, 100 kVp, 200 kVp, 250 kVp (Siemens, Concord, CA) and 6MV-linac (Varian 600 C linear accelerator, Varian Associates, Palo Alto, CA, USA). Dose rates at the surface of the cell culture in the case of kVp X-rays were measured according to the dose protocol of TRS277^18^. The dose rates for 60 kVp, 100 kVp, 200 kVp and 250 kVp X-rays were 0.42 Gy/min, 0.86 Gy/min, 1.25 Gy/min and 1.26 Gy/min, respectively. It should be noted that the dose attenuation can be negligible in culture medium (the depth is 1 mm as water equivalent) for all types of X-rays. As for 6 MV linac X-rays, the dose rate was measured according to Japanese Standard Dosimetry 12^19^. The dose rates at the isocenter for in-field 6 MV X-rays at 1 cm, 3 cm, 5 cm and 10 cm depth were 4.91 Gy/min, 4.55 Gy/min, 4.44 Gy/min and 3.75 Gy/min, respectively, whilst the dose rate for out-of-field 6 MV X-rays at 10 cm depth was 0.10 Gy/min. For the MV X-rays irradiation, the cell culture dishes were fully filled with cell culture medium. All of the dose rates for the both kV X-rays and MV X-rays are based on water kerma. The absorbed dose of 1.0 Gy was delivered to cells for all types of X-rays. Each experiment was performed at room temperature.

### Detection of DNA-DSBs by γ-H2AX foci formation assay

The number of initial DNA-DSBs per nucleus after exposure to 1.0 Gy was measured by means of γ-H2AX foci formation assay. At 30 min after irradiation, the irradiated cells were fixed in 4% paraformaldehyde solution for immunofluorescence microscopy while in 70% ethanol for flow cytometry and kept with ice or in a −20 °C freezer. The cells were rinsed with PBS(−) and permeabilized in ice-cold 0.2% Triton X-100 in PBS for 5 min, and blocked with a solution of 1% BSA in PBS for 30 min. A primary anti-body γ-H2AX diluted by a 1% BSA in PBS was then fed and kept at 4 °C overnight for immunofluorescence microscopy while at room temperature for 2 h for flow cytometry. After rinsing with a solution of 1% BSA-containing PBS three times, Alexa Fluor 594- or 488-conjugated goat-anti-rabbit (Molecular Probes, Invitrogen, Japan) diluted by a solution of 1% BSA-containing PBS was fed and kept for 2 h for immunofluorescence microscopy while for 30 min for flow cytometric analysis in the dark at room temperature. We then measured the intensity of γ-H2AX per cell nucleus by a High Standard all-in-one fluorescent microscope (model BZ-9000; Keyence, Osaka, Japan) and by an Attune acoustic focusing flow cytometer (Applied Biosystems by Life Technologies TM). In the case of microscopy, the number of γ-H2AX foci per nucleus was counted up to above 143 cells by using Image J software. The foci intensity detected by the flow cytometer was converted to the number of DSBs based on the counted number by microscopy as reported previously^16^. We have checked the linearity of the foci intensity with respect to the number of DSBs and confirmed that the relation between the intensity and the number is positively correlated.

### Relative biological effectiveness (RBE) at the endpoint of DNA-DSB

To evaluate the dependency of photon energy on biological effects from the γ-H2AX foci formation assay, we adopted the relative biological effectiveness at the endpoint for DNA-DSB (RBE_DSB_)^20,21^. The number of DNA-DSBs per nucleus is proportional to the absorbed dose^20,22^, and RBE is given by the ratio of the radiation type to standard radiation such as 200 kVp X-rays. Thus, the RBE_DSB_ can be obtained from the ratio of the number of DNA-DSBs per nucleus at 1.0 Gy of irradiation with a certain type of photon beam to that with 200 kVp X-rays (standard radiation) as follows,

3 $${{\rm{RBE}}}_{{\rm{DSB}}}=\frac{{{\rm{DSB}}}_{{\rm{subject}}{\rm{X}} \mbox{-} \mathrm{rays}}}{{{\rm{DSB}}}_{{\rm{200}}{\rm{kVp}}{\rm{X}} \mbox{-} \mathrm{rays}}}.$$

Based on the dose-mean lineal energy (*y*_*D*_) and RBE_DSB_ defined above, we evaluated the impact of photon energy dependency on biological effects.

## Results

### Dose-mean lineal energy for mono-energetic electrons and X-rays

**Figure 3:**
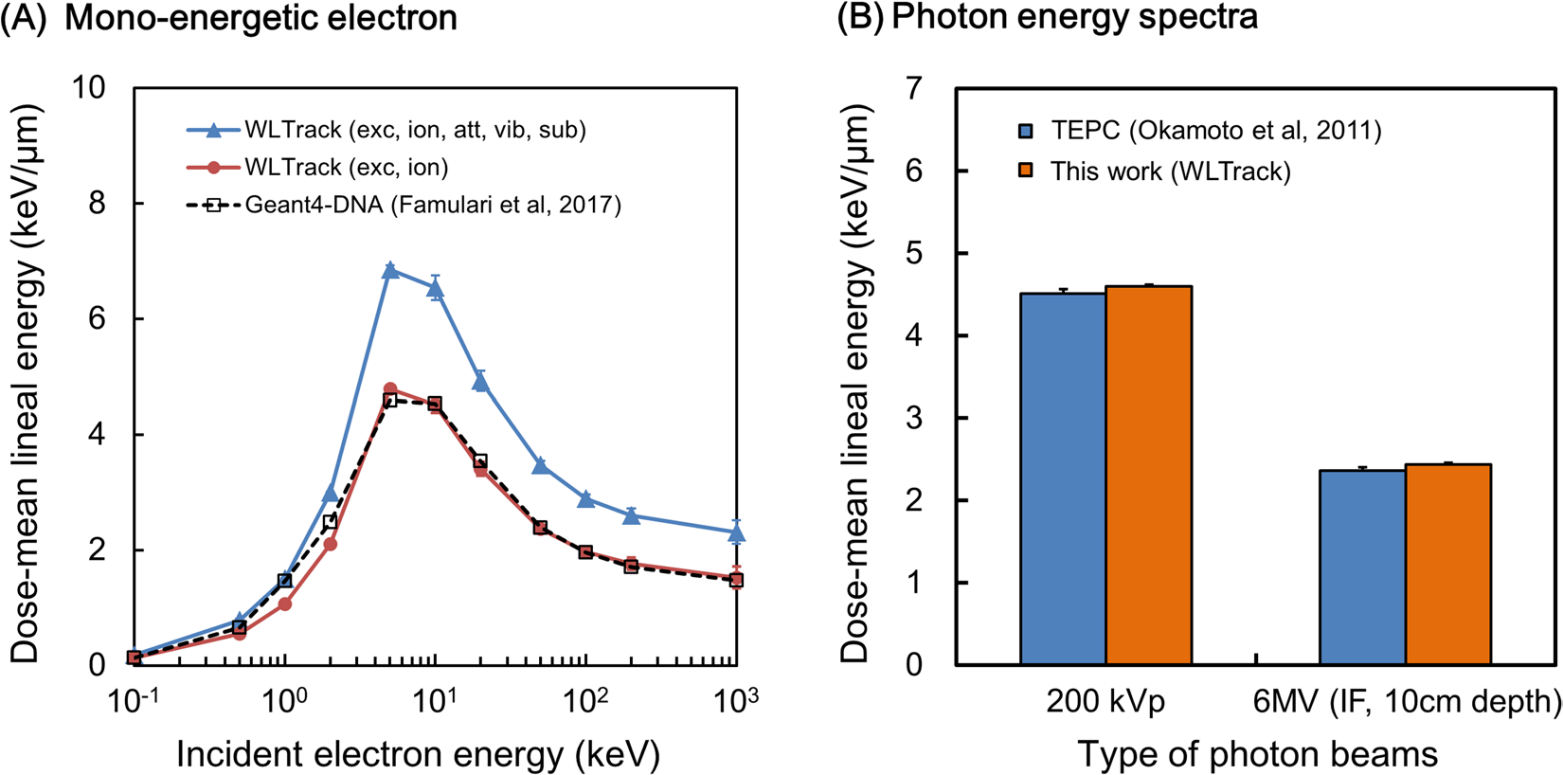
Comparison of *y*_*D*_ values between this work and reference data. (**A**) Is for electrons in the range of kinetic electron energy from 0.1 to 1000 keV and (**B**) for two photon spectra of 200 kVp and 6 MV X-rays (at 10 cm depth from surface in in-field region). In (**A**), we assumed two physical conditions: one is for considering all electron collision processes, excitation (electronic), ionization, attachment, vibrational excitation and elastic scattering (blue triangle), and the other for only three processes of excitation (electronic), ionization, and elastic scattering (red circle). The results of GEANT4-DNA (option5) reported by Famulari *et al*.^3^ are plotted as reference (white square). The results of WLTrack (exc, ion, elas) exhibit approximately 70% lower than those of WLTrack (exc, ion, att, vib, sub, elas), whilst the *y*_*D*_ values calculated by WLTrack (exc, ion, att, vib, sub, elas) for both photon spectra agree well with the measured data by tissue equivalent proportional counter (TEPC) reported by Okamoto *et al*.^15^. Standard errors of all calculation were based on three independent calculations.

To examine the validity of our code for calculating the lineal energy *y* for photons, we first obtained the electron dose-mean lineal energy *y*_*D*_ as a function of initial electron energy in keV. Figure 3 shows the *y*_*D*_ value of mono-energetic electrons calculated for two cases: one for considering all events (denoted by closed triangles in Fig. 3A) as case 1, and the other for considering only three events, ionization, electronic excitation, and elastic scattering, (denoted by closed circles) as case 2. The open squares in Fig. 3 represent the reference data by Famulari, *et al*.^3^. The *y*_*D*_ values calculated for case 1 were approximately 1.43 times larger than the reference values. Because the *y*_*D*_ values in the ref. ^3^ were calculated based on the ionization and electronic excitation events as energy transfer processes, we added the *y*_*D*_ values calculated for case 2 for comparison (energy transfer by elastic scattering is negligible). The results for case 2 are in good agreement with the reference data and suggest that the energy deposition by ionization and electronic excitation accounts for about 70% of the total energy deposition.

The *y*_*D*_ values for a variety of photon spectra were also calculated by accumulating the contribution of electron processes, where the energy spectra of the electrons produced by photoelectric absorption and Compton scattering processes were computed by PHITS code^14^. The *y*_*D*_ values for 200 kVp X-rays as a standard radiation and 6 MV linac X-rays at 10 cm depth from the surface are listed in Table 1 in comparison with the data measured by Tissue Equivalent Proportional Counter (TEPC)^15^. As shown in Fig. 3B, the *y*_*D*_ values calculated for 200 kVp and 6MV X-rays are in very good agreement with the TEPC data by Okamoto, *et al*.^15^. From this benchmark test, it was deemed that the in-house Monte Carlo code for electrons “WLTrack” and the sampling technique are precise enough to calculate the microdosimetric quantities for electrons and photons.

**Table 1:** Calculated dose-mean lineal energy *y*_*D*_ and measured RBE_DSB_ for various X-ray spectra.

Type	Energy of X-rays (irradiation conditions)	*y*_*D*_ (keV/μm) this work	RBE_DSB_
Standard	200 kVp (0.5 mm Cu + 0.5 mm Al)	4.60 ± 0.02	1.00 ± 1.49
Therapeutic X-rays	6 MV-linac (10 cm depth) in-field (field size:10 × 10 cm^2^)	2.44 ± 0.02	0.85 ± 1.33
	6 MV-linac (5 cm depth) in-field (field size:10 × 10 cm^2^)	2.47 ± 0.03	0.76 ± 1.04
	6 MV-linac (1 cm depth) in-field (field size:10 × 10 cm^2^)	2.45 ± 0.02	0.73 ± 0.99
	6 MV-linac (10 cm depth) out-of-field (field size:10 × 10 cm^2^)	3.00 ± 0.01	0.85 ± 1.26
Diagnostic X-rays	60 kVp (2 mm Al)	4.39 ± 0.02	1.18 ± 1.52
	100 kVp (1.0 mm Al)	4.53 ± 0.01	1.17 ± 1.62
	250 kVp (No filtraction)	4.45 ± 0.01	1.39 ± 1.78

**Figure 4:**
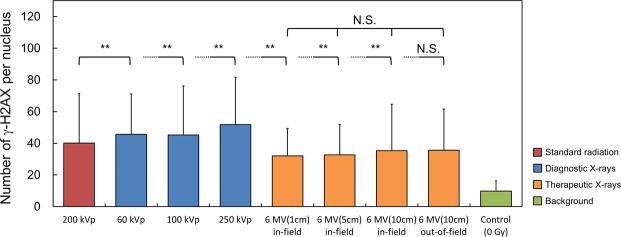
Numbers of DSBs per nucleus for a variety of photon irradiations. The number of DSBs was obtained by γ-H2AX foci formation assay. Error bar indicates the standard deviation in the number of DSBs per nucleus. The number of cell analyzed for each condition is 143–228 (by microscopy) or 2416–68673 (by flow cytometry). Significant differences are observed (by the Tukey-Kramer test) for the cases of lower energy X-rays (60, 100, 250 kVp) and in-field irradiation with 6MV-linac X-rays (**p < 0.01). In general, the numbers of foci for diagnostic kilo-voltage X-rays are greater than those of therapeutic mega-voltage X-rays.

The RBE_DSB_ was obtained as the ratio of the number of γ-H2AX foci per nucleus at 30 min (immediately) after irradiation (obtained by subtracting the background number) for each X-ray to that for 200 kVp X-ray (0.5 mm Cu + 0.5 mm Al) as the standard radiation^8,16,17^. The s.d. values of the *y*_*D*_ were evaluated based on three independent calculations. The s.d. of the RBE_DSB_ was derived from the error propagation of the s.d. in the number of γ-H2AX foci per nucleus shown in Fig. 4.

Based on the validation above, we calculated the *y*_*D*_ values for 60 kVp, 100 kVp, 250 kVp and 6 MV linac X-rays (at various depths from the surface within an in-field region and at 10 cm depth from the surface in an out-of-field region at 10 cm away from isocenter) considering their spectra. The calculated *y*_*D*_ values for X-rays with energies, 60–250 keV (diagnostic) and 6 MeV (therapeutic), are summarized in the first and second columns of Table 1. From the calculation results in Table 1 (third column), the *y*_*D*_ values for diagnostic X-rays with 60 kVp and 100 kVp are slightly higher than that for the standard radiation of 200 kVp, whilst the value for 6 MV linac X-rays is lower than that for 200 kVp X-rays. *y*_*D*_ represents the concentration of energy deposition along radiation particle tracks. Thus, the present result indicates that the energy deposition per track length after irradiation by diagnostic X-rays (with relatively low energy) is higher than that by therapeutic X-rays (with relatively high energy).

### Dependency of X-ray’s energy on initial DNA-DSB induction and RBE_DSB_

To evaluate the impact of photon energy dependency on biological effects, we next measured the number of DSBs per cell nucleus 30 min after irradiation of 1.0 Gy by means of γ-H2AX foci formation assay. The number of γ-H2AX foci per nucleus measured in this study is shown in Fig. 4. The mean number of DSBs per nucleus after 1.0 Gy irradiation with diagnostic X-ray energy (60–250 kVp X-rays) ranged from 30.2 to 41.9 whilst that after the irradiation with therapeutic X-ray energy (6 MV linac X-ray) is from 22.2 to 25.9 as listed in Table 1. The yield of initial DNA-DSB induction for kilo-voltage X-rays is higher than that for mega-voltage X-rays. Focusing on the dependency of depth from the surface under the 6MV X-ray irradiation (Fig. 2B,C), there is no significant difference among 1.0 cm, 5.0 cm, 10.0 cm depth in in-field region and 10.0 cm depth in the out-of-field region (Fig. 4).

**Figure 5:**
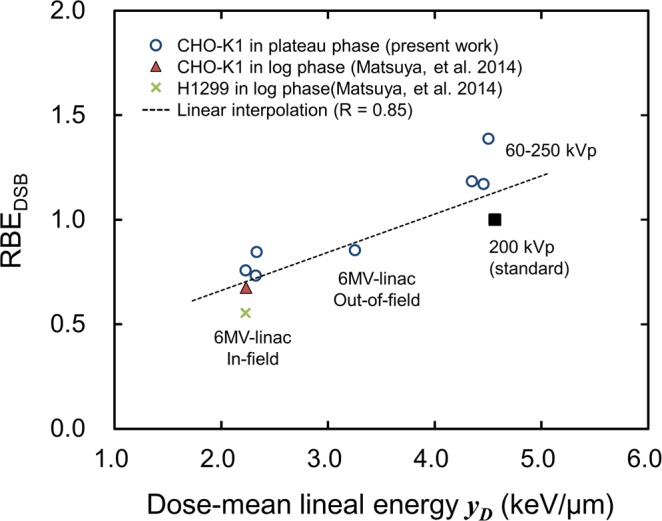
Relation between the *y*_*D*_ values calculated for various types of X-rays and measured RBE_DSB_. Whilst the symbol of open circle in blue represents new data in this study, the other symbols indicate data taken from a previous report^7^ for comparison. The RBE_DSB_ tends to be higher as mean energy of X-rays is lower.

Next, we calculated the relative biological effectiveness (RBE) at the endpoint of DNA-DSBs (RBE_DSB_). The number of radiation-induced γ-H2AX foci measured in this study (Table 1) was converted to the RBE_DSB_ according to Eq. (3). The RBE_DSB_ from the experiments is listed in the fourth row of Table 1. In the same manner as the measured foci number, the RBE_DSB_ for kilo-voltage X-rays is higher than that for mega-voltage X-ray. For instance, the RBE_DSB_ for the kilo-voltage X-rays and mega-voltage X-rays ranged from 1.00 to 1.39 and from 0.73 to 0.85, respectively. By adding experimental RBE_DSB_ results, we determined the relationship between the calculated *y*_*D*_ and RBE_DSB_ as shown in Fig. 5, where the RBE_DSB_ monotonically increases as the *y*_*D*_ value increases.

## Discussion

### Concentration of energy deposition along the electron track and DNA damage

The *y*_*D*_ values for various mono-energetic electrons and photons were evaluated from the viewpoints of the Monte Carlo simulation and DNA damage detection. The experimental data associated with DNA damage induction showed that the DNA*-*DSBs yield depends on X-ray energy (Table 1).

In terms of physical characteristics, the concentration of energy deposition along electron tracks generated by incident photons is relatively high at the end of the track^13,23^. Irradiation by high energy X-rays on biological tissue generates high energy secondary electrons which conduce to lower the *y*_*D*_ value (Fig. 3). For this reason, the probability of local energy deposition for high energy (mega-voltage) X-rays is smaller than that for low energy (kilo-voltage) X-rays. Collectively, both simulation and biological experiment demonstrate that diagnostic X-rays have higher biological effects than therapeutic X-rays (Fig. 4). This tendency is in good agreement with the results by other previous reports^5,24^, suggesting that the secondary electrons generated by lower energy X-rays are more effective to yield the DNA damage than those by higher energy X-rays. The present work provides a quantitative relation between the microdosimetry and yield of DNA-DSB (as shown in Fig. 3 and Table 1).

The *y*_*D*_ value of the out-of-field 6 MV-linac X-rays is slightly higher than that of in-field X-rays, which suggests that scattered X-rays with lower energy from the collimator contributes to the increase of the local energy deposition. However, the experimental number of DNA-DSBs induced by out-of-field 6 MV-linac X-rays is not significantly larger than that by in-field X-rays. As for the exposure to out-of-field 6 MV linac X-rays at 0.10 Gy/min, a relatively long dose-delivery time is needed in comparison with in-field exposure, e.g., 10 min. The DNA damage repair by virtue of non-homologous end joining (NHEJ)^25,26^ as a quick repair process may function during irradiation^27^, which can diminish the number of γ-H2AX foci after the irradiation by out-of-field 6 MV linac X-rays. Considering the rate constant for DNA repair of CHO-K1 cells in plateau phase (0.704 [h-1]), the measured radiation-induced γ-H2AX foci number per nucleus (25.9 per nucleus) can be correlated to be 27.5 per nucleus, based on the microdosimetric-kinetic (MK) model for continuous irradiation^17^. To evaluate the precise initial DNA-DSB induction yield for out-of-field 6 MV linac X-rays, further experimental investigation with the use of acute photon irradiation with about 3.0 keV/μm as the *y*_*D*_ value (e.g., cesium-137 γ -rays (*y*_*D*_ = 2.90 keV/μm^7^) may be necessary.

### Cross sections for ionization and excitation processes of electrons

The validity of our simulation code was confirmed as shown in Fig. 3B. The slight discrepancy between the calculated *y*_*D*_ value and the measured value is partly attributable to the differences of physics models including cross sections (such as ionization and excitation) and the energy allocated to ejected (secondary) electrons after ionization events, etc. Comparing the cross sections used in WLTrack^13^ with those in Geant4-DNA^28–30^, the electronic excitation cross section in Geant4-DNA (Emfietzoglou model)^30^ is larger than that in WLTrack for kinetic energy of electrons up to 30 eV. In contrast, the ionization cross section in WLTrack is larger than that in Geant4-DNA (Born model)^30^ for energy above about 80 eV. The calculated result for *y*_*D*_ comprises comprehensive contributions from these cross sections. In either code, however, each cross section has a peak at a low energy region (10–30 eV for excitation, 80–200 eV for ionization), which contributes to the maximum local energy deposition around at the end of the electron tracks. Thus, low initial energy electrons have higher probabilities (per track) to induce local damage to intracellular structures than high initial energy electrons. The *y*_*D*_ value enables us to quantize this situation. The collision cross sections of electrons are crucial to calculate the *y*_*D*_ value by track simulation. However, it is very difficult in general to determine the individual cross sections of electrons colliding with water molecules (particularly in liquid phase) because plural types of collision processes (e.g., electronic excitation, vibrational excitation, attachment) can occur at the same electron energy below several tens of eV^30^. Meanwhile, it has been reported in past decades that low energy electrons with below 20 eV also has an impact on DNA single-strand breaks and DNA-DSBs induction^31–33^. Thus, the update for the electron collision cross sections should be required for investigating the mechanisms on how a low-energy electron induces a DNA damage.

## Conclusion

In this study, we evaluated the biological effectiveness of X-ray beams from a Monte Carlo simulation (of photons and electrons) and a biological experiment to detect DNA-DSBs. It was revealed that the biological effectiveness in terms of DNA damage depends on incident X-ray energy. The effectiveness of diagnostic X-rays (60–250 kVp) was shown to be greater than that of therapeutic X-rays (linac 6 MV) even at the same absorbed dose. The calculation of the dose-mean lineal energy (*y*_*D*_) indicates that the *y*_*D*_ value can be an index to measure the biological effectiveness corresponding to the X-ray energy. This finding suggests that the radiation weighting factor (*w*_*R*_) for photons and electrons should not be unity but be defined as a function of electron energy. Because the electron collision cross sections are essential to calculate the *y*_*D*_ value, the update of cross sections is necessary to increase the accuracy of the calculated result.

## References

[1] Nikjoo, H., Uehara, S. & Emfietzoglou, D. Interaction of photons with matter. In: Nikjoo H, Uehara S, Emfietzoglou D. *Interaction of radiation with matter*. Boca Raton: *CRC Press*; 89–102 (2012).

[2] Wouters, B. G. & Begg, A. C. Irradiation-induced damage and the DNA damage response. In: Joiner, M. & van der Kogel, A. J. (eds). *Basic Clinical Radiobiology*. London: Edward Arnold; p. 11–26 (2009).

[3] Famulari G, Pater P, Enger SA (2017). Microdosimetry calculations for monoenergetic electrons using Geant4-DNA combined with a weighted track sampling algorithm. Phys. Med. Biol..

[4] ICRP Recommendations of the ICRP–ICRP report No. 60. In: Annals of ICRP international commission on radiological protection; 1–201 (1991). 2053748

[5] Beyreuther E, Lessmann E, Pawelke J, Pieck S (2009). DNA double-strand break signalling: X-ray energy dependence of residual co-localised foci of γ-H2AX and 53BP1. Int. J. Radiat. Biol..

[6] Nikjoo H, Lindborg L (2010). RBE of low energy electrons and photons. Phys. Med. Biol..

[7] Matsuya Y (2014). Quantitative estimation of DNA damage by photon irradiation based on the microdosimetric-kinetic model. J. Radiat. Res..

[8] Hall, E. J. & Giaccia, A. J. Linear Energy Transfer and Relative Biological Effectiveness. In: Hall, E. J. & Giaccia, A. J. (eds). *Radiobiology for the Radiologist*. Lippincott Williams & Wilkins, p 106-116 (2006).

[9] Joiner, M. C. Linear energy transfer and relative biological effectiveness. In: Michael, J. & van der Kogel, A. J. (eds). *Basic Clinical Radiobiology*, 4th edn. London: Hodder Arnold, p 68–77 (2009).

[10] ICRU. Microdosimetry. Report 36. International Commission on Radiation Units and Measurements. Bethesda: MD (1983).

[11] Nikjoo H, O’Neill P, Wilson WE, Goodhead DT (2001). Computational Approach for Determining the Spectrum of DNA Damage Induced by Ionizing Radiation. Radiat. Res..

[12] Friedland, W., Bernhardt, P., Jacob, P., Parezke, H. G. & Dingfelder, M. Simulation of DNA damage after proton and low LET irradiation. *Radiat. Prot. Dosimetry* **99**(1–4), 99–102 (2002). 10.1093/oxfordjournals.rpd.a006848 12194370

[13] Date H, Sutherland KL, Hasegawa H, Shimozua M (2007). Ionization and excitation collision processes of electrons in liquid water. Nucl. Instrum. Methods Phys. Res. B.

[14] Sato T (2018). Features of Particle and Heavy Ion Transport code System (PHITS) version 3.02. J. Nucl. Sci. Technol..

[15] Okamoto H (2011). Relation between Lineal Energy Distribution and Relative Biological Effectiveness for Photon Beams according to the Microdosimetric Kinetic Model. J. Radiat. Res..

[16] Mori R, Matsuya Y, Yoshii Y, Date H (2018). Estimation of the radiation-induced DNA double- strand breaks number by considering cell cycle and absorbed dose per cell nucleus. J. Radiat. Res..

[17] Matsuya Y (2018). Investigation of dose-rate effects and cell-cycle distribution under protracted exposure to ionizing radiation for various dose-rates. Scientific Reports.

[18] International Atomic Energy Agency (IAEA). Absorbed dose determination in photon and electron beams. An International Code of Practice. Technical Reports Series No. 277, Vienna (1987).

[19] JSMP. Standard dosimetry of absorbed dose in external beam radiotherapy (Standard Dosimetry 12). Tsusho Sangyo Kenkyu Sha, Tokyo (2012).

[20] Gerelchuluun A (2011). Induction of *in situ* DNA double-strand breaks and apoptosis by 200 MeV protons and 10 MV X-rays in human tumour cell lines. Int. J. Radiat. Biol..

[21] Franken NAP (2014). Comparison of RBE values of high- LET a-particles for the induction of DNA-DSBs, chromosome aberrations and cell reproductive death. Radiat. Oncol..

[22] Macphail SH (2003). Expression of phosphorylated histone H2AX in cultured cell lines following exposure to X-rays. Int. J. Radiat. Biol..

[23] Yoshii Y, Sutherland KL, Date H (2011). Electron track analysis for damage formation in bio-cells. Nucl. Instrum. Methods Phys. Res. B.

[24] Freneau A (2018). Relation between DNA double-strand breaks and energy spectra of secondary electrons produced by different X-ray energies. Int. J. Radiat. Biol..

[25] Hawkins RB, Inaniwa T (2013). A microdosimetric-kinetic model for cell killing by protracted continuous irradiation including dependence on LET I: repair in cultured mammalian cells. Radiat. Res..

[26] Hufnagl A (2015). The link between cell-cycle dependent radiosensitivity and repairpathways: A model based on the local, sister-chromatid conformationdependent switch between NHEJ and HR. DNA Rep..

[27] Matsuya Y, Kimura T, Date H (2017). Markov chain Monte Carlo analysis for the selection of a cell-killing model under high-dose-rate irradiation. Med. Phys..

[28] Bernal MA (2015). Track structure modeling in liquid water: A review of the Geant4-DNA very low energy extension of the Geant4 Monte Carlo simulation toolkit. Physica Medica.

[29] Incerti S (2010). Comparison of GEANT4 very low energy cross section models with experimental data in water. Med. Phys..

[30] Incerti S (2010). The Geant4-DNA project. Int. J. Mod. Simul. Scien. Comput..

[31] Boudaїffa B, Cloutier P, Hunting D, Huels MA, Sanche L (2000). Resonant Formation of DNA Strand Breaks by Low-Energy (3 to 20 eV) Electrons. Science.

[32] Simons J (2006). How Do Low-Energy (0.1–2 eV) Electrons Cause DNA-Strand Breaks?. Acc. Chem. Res..

[33] Martin F (2004). DNA Strand Breaks Induced by 0–4 eV Electrons: The Role of Shape Resonances. Phys. Rev. Lett..

